# Invasive haemodynamic validation of an artificial intelligence echocardiographic tool for detecting heart failure with preserved ejection fraction

**DOI:** 10.1093/eschf/xvag052

**Published:** 2026-04-07

**Authors:** Ryan Sachar, Maria Latz, Tess Allan, John E Blair, Gene Kim, Jonathan Grinstein, Gary Woodward, Roberto Lang, Mark N Belkin

**Affiliations:** Section of General Internal Medicine, University of Chicago Medicine, Chicago, IL, USA; Section of Cardiology, University of Chicago Medicine, 5841 South Maryland Avenue, MC 6092 Chicago, IL 60637, USA; Division of Cardiology, Duke University Medical Center, Durham, NC, USA; Division of Cardiology, University of Washington, Seattle, WA, USA; Section of Cardiology, University of Chicago Medicine, 5841 South Maryland Avenue, MC 6092 Chicago, IL 60637, USA; Section of Cardiology, University of Chicago Medicine, 5841 South Maryland Avenue, MC 6092 Chicago, IL 60637, USA; Ultromics Ltd, Oxford, UK; Section of Cardiology, University of Chicago Medicine, 5841 South Maryland Avenue, MC 6092 Chicago, IL 60637, USA; Section of Cardiology, University of Chicago Medicine, 5841 South Maryland Avenue, MC 6092 Chicago, IL 60637, USA

**Keywords:** Heart failure with preserved ejection fraction, Artificial intelligence, Echocardiography, Right heart catheterization, Pulmonary capillary wedge pressure, Diagnostic screening tool

## Abstract

**Introduction:**

To externally validate an FDA-approved artificial intelligence (AI) tool for detecting heart failure with preserved ejection fraction (HFpEF) using echocardiographic video clips, comparing its performance to invasive haemodynamic criteria in a real-world referral cohort with unexplained dyspnoea.

**Methods:**

We retrospectively analysed 47 patients who underwent transthoracic echocardiography (TTE) and right heart catheterization (RHC), including 28 with both rest and exercise haemodynamics. The AI model evaluated apical 4-chamber video data and classified outputs as HFpEF, no HFpEF, or non-diagnostic, without any other clinical data. The primary outcome was invasively defined HFpEF: pulmonary capillary wedge pressure (PCWP) ≥15 mmHg at rest or ≥25 mmHg with exercise. Secondary analysis used a PCWP/cardiac output (CO) slope >2 mmHg/L/min. We assessed sensitivity, specificity, positive predictive value (PPV), and negative predictive value (NPV).

**Results:**

Among patients with exercise RHC (*n* = 28), 71% met haemodynamic HFpEF criteria. The AI tool demonstrated sensitivity of 30%, specificity of 88%, PPV of 86%, and NPV of 33%. Using the PCWP/CO slope (*n* = 25), specificity and PPV were 100%, sensitivity 27%, and NPV 16%. AI-positive patients had significantly higher resting PCWP (20 vs 15 mmHg, *P* = .029), mean PA pressure (29 vs 24 mmHg, *P* = .02), and PVR (2.1 vs 1.3 WU, *P* = .002). In patients with indeterminate H_2_FPEF scores (*n* = 25), the AI correctly identified 80% of those with invasively confirmed HFpEF. Model performance was consistent across TTE-RHC intervals <365 and <90 days.

**Conclusions:**

This AI model demonstrated high specificity and positive predictive value (PPV) for detecting HFpEF, reliably identifying patients with more advanced haemodynamic abnormalities. Its performance remained robust across variable intervals between transthoracic echocardiography (TTE) and right heart catheterization (RHC), and in patients with indeterminate clinical scores. Due to limited sensitivity, the tool is best utilized to enrich identification of patients with clearly abnormal haemodynamics rather than to exclude HFpEF, particularly in early or borderline cases. While broader use as a screening tool is promising, prospective validation studies are necessary to confirm its utility in general populations.

## Graphical Abstract

**Graphical Abstract:**
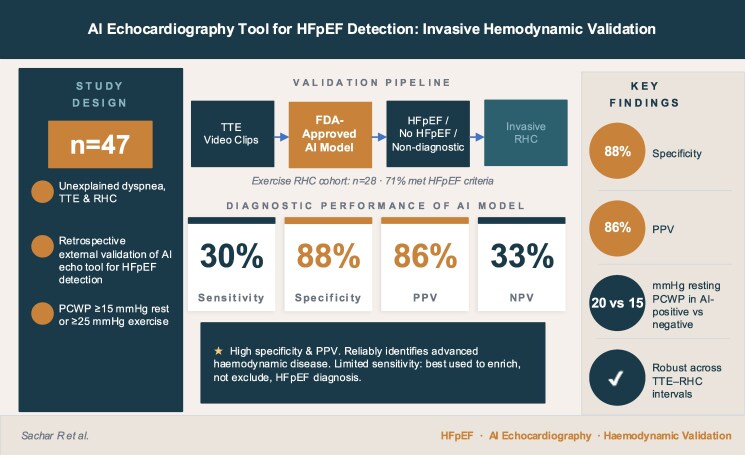

## Introduction

Heart failure with preserved ejection fraction (HFpEF) accounts for approximately half of all heart failure cases in the USA, with incidence rising alongside an ageing population. At age 45, the lifetime risk of developing HFpEF is estimated at 10%, and among patients who have been hospitalized, the 5-year mortality rate approaches 75%.^1–5^ Despite its growing prevalence and poor outcomes, HFpEF remains challenging to diagnose in routine practice, particularly due to the heterogeneous clinical presentations and the high burden of comorbidities in affected patients.

Assessment of diastolic function using transthoracic echocardiography (TTE) is a cornerstone of non-invasive evaluation of HFpEF, yet current imaging guidelines often underdiagnose HFpEF and frequently categorize patients into indeterminate risk groups.^6–11^ To address these gaps, scoring tools such as the H_2_FPEF and HFA-PEFF scores have been developed to streamline diagnostic pathways. However, both tools have notable limitations, including a high rate of false negatives and indeterminate classifications, contributing to underdiagnosis.^12–14^ When scores are non-diagnostic or indeterminate, current algorithms recommend downstream testing, such as exercise right heart catheterization (RHC) or stress echocardiography, for confirmation. However, neither of these is routinely performed in the real-world, further contributing to the underdiagnosis of HFpEF.

In response to these limitations, a novel artificial intelligence (AI) algorithm was recently developed using a 3D convolutional neural network trained on 6756 apical 4-chamber, 30-frame TTE video clips from patients with expert-adjudicated HFpEF or non-HFpEF phenotypes. HFpEF was defined using clinical criteria (preserved ejection fraction, signs/symptoms of heart failure, and supportive biomarkers/imaging) but did not require invasive haemodynamic testing. The model operates on video data alone, requires no additional clinical input, and was trained and validated using data from the Mayo Clinic. Model outputs were classified as HFpEF, no HFpEF, or non-diagnostic. It demonstrated high diagnostic performance (AUROC 0.95–0.97), outperforming traditional risk scores with outputs predictive of mortality risk. However, only a minority of patients in the training dataset underwent invasive haemodynamic testing, and none underwent exercise right heart catheterization.^15^

While the model has been previously validated in multiple independent studies, including work by Akerman et al. and Karnik *et al*., those studies primarily relied on clinical and imaging-based criteria for HFpEF rather than invasive haemodynamic measurements.^16,17^ In this study, we externally validated this AI-based echocardiographic screening tool using invasive haemodynamic data in a real-world referral cohort undergoing evaluation for unexplained dyspnoea.

## Methods

**Figure 1:**
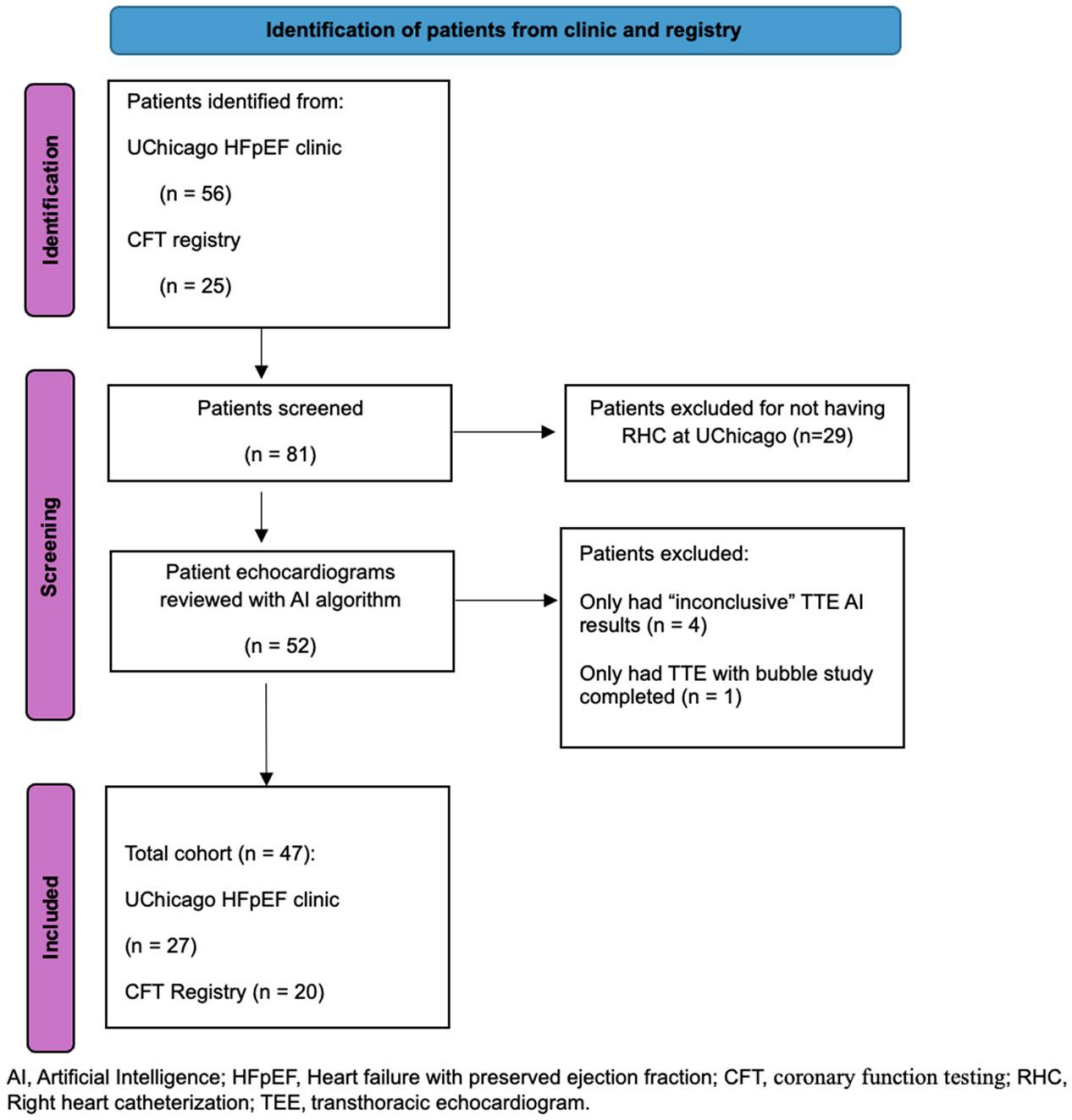
Patient inclusion flow diagram. The PRISMA flow diagram illustrates the process of identifying patients for study inclusion. Two initial cohorts—patients from the University of Chicago (UChicago) heart failure with preserved ejection fraction (HFpEF) clinic and those from the coronary function testing (CFT) registry—were merged. Patients who had not undergone right heart catheterization (RHC) at UChicago were excluded. Additional exclusions were made for inconclusive or incomplete transthoracic echocardiograms (TTEs), resulting in a final analytic cohort of 47 patients

We retrospectively included patients with TTE imaging completed at our institution. This retrospective study was approved by the institutional review board with a waiver of consent. Two cohorts of patients were included in this retrospective analysis: (i) the University of Chicago HFpEF clinic and (ii) patients previously enrolled in a prospective registry of patients that underwent coronary function testing (CFT) as part of their evaluation for HFpEF (*Figure 1*). The University of Chicago HFpEF clinic includes patients referred for evaluation and management of suspected HFpEF, defined as signs and symptoms of HF and an LVEF ≥50%; patients with end-stage renal disease or advanced pulmonary disease requiring continuous ambulatory oxygen were excluded. The CFT registry enrolled from January 2015 to January 2021, and it has been previously described.^18^ In brief, this was a prospective registry of patients with suspected HFpEF undergoing CFT to assess for coronary microvascular dysfunction. Patients in this cohort underwent RHC at baseline ± exercise in addition to coronary angiography and CFT for assessment of flow reserve, index of microvascular resistance, and response to acetylcholine to assess for coronary microvascular dysfunction and coronary vasospasm. This database initially evaluated patients with resting RHC alone, but as the recumbent bicycle ergometer became available to the institution in January 2017, all patients were evaluated with resting and exercise RHC. All exercise RHC was done in the supine position.

The primary outcome was haemodynamically defined HFpEF, based on a pulmonary capillary wedge pressure (PCWP) ≥ 15 mmHg at rest or ≥25 mmHg during exercise. Secondary outcomes included HFpEF defined by PCWP/cardiac output (CO) slope >2 mmHg/L/min, a metric increasingly recognized for its sensitivity in detecting early or occult HFpEF.^19^ Additional subgroup analyses included performance among patients with an indeterminate H_2_FPEF score, patients with resting RHC data only, and across different time intervals between TTE and RHC.

### Haemodynamic testing protocol for patients in CFT cohort

Following measurements of resting haemodynamics, patients first had their legs placed in the supine ergometer pedals for 3 min after which pulmonary artery (PA) and right atrial (RA) pressures were measured. Supine cycle ergometry exercise was then initiated at 20 W and increased in 20-W increments every 3 min until patient-limited maximal effort was achieved. Haemodynamic pressures were recorded at each stage of exercise, while thermodilution cardiac output was measured at peak workload only.

### Statistical analysis

Statistical analysis included calculation of diagnostic performance metrics: sensitivity, specificity, positive predictive value (PPV), negative predictive value (NPV), and likelihood ratios. Haemodynamic values were compared between AI ‘positive’ and ‘negative’ patients using Wilcoxon rank-sum tests.

## Results

A total of 47 patients were included in the final analysis, 28 of whom had both resting and exercise RHC. The median age was 62 years, 83% of patients were women, and 79% identified as Black. Atrial fibrillation was present in 21% of the cohort, and the median BMI was 33.7 kg/m^2^. Additional baseline characteristics are presented in *Table 1*. Among the total cohort, 18 were classified as AI ‘positive’ and 29 as ‘negative.’ Four patients were excluded due to indeterminate TTE studies, corresponding to a 5% exclusion rate.

**Table 1:** Clinical and demographic characteristics of patients in the full cohort and AI ‘positive’ and ‘negative’ cohorts

Baseline characteristics	Full cohort (*n* = 47)	AI positive cohort (*n* = 18)	AI negative cohort (*n* = 29)	*P*-value
**Sex**				
** Women**	39 (83%)	16 (89%)	23 (79%)	.7
** Men**	8 (17%)	2 (11%)	6 (21%)	.157
**Race**				
** White**	7 (14.9%)	1 (6%)	6 (20%)	.23
** Black**	37 (78.7%)	14 (78%)	23 (79%)	1.00
**Age (years)**	62	66	60	.**04**
**Body mass index (kg/m^2^)**	33.7	33.6	33.7	.97
**No. with Afib**	10 (21.2%)	7 (39%)	3 (10%)	.05
**NT-proBNP (ug/mL)**	173	451	117	**<**.**01**
**LVEF (%)**	62	61	62	.68
**H2FPEF score**	5	6	4	**<**.**01**
**HFA-PEFF score**	3	5	2	**<**.**01**
**Lifetime AHFH**	0	1	0	.**01**

Values are presented as median or *n* (%). The *P*-values reported are based on a Wilcoxon test for non-normally distributed continuous variables, and a χ^2^ test for categorical variables. Bolded values indicate statistical significance at P < 0.05.

AHFH, acute heart failure hospitalizations; Afib, Atrial fibrillation; H2FPEF, heart failure with Preserved Ejection Fraction Score; HFA-PEFF, Heart Failure Association Pre-test assessment, Echocardiography and natriuretic peptide, Functional testing, Final aetiology; LVEF, left ventricular ejection fraction; No., number; NT-proBNP, N-terminal pro b-type natriuretic peptide.

### Invasive haemodynamic analysis

Among the 28 patients who underwent both resting and exercise RHC, 20 (71%) met HFpEF criteria based on either a resting PCWP ≥15 mmHg or an exercise PCWP ≥ 25 mmHg. In this subgroup, the AI model identified 7 patients as ‘positive,’ 6 of whom (86%) were true positives. Among the 21 ‘negative’ patients, 14 (67%) met invasive HFpEF criteria. This yielded a sensitivity of 30%, specificity of 88%, PPV of 86%, NPV of 33%, positive likelihood ratio (LR+) of 2.4, and negative likelihood ratio (LR−) of 0.8.

In the 25 patients (89%) with complete rest and exercise PCWP and cardiac output data, 22 (88%) met HFpEF criteria defined by a PCWP/CO slope > 2 mmHg/L/min. Of the 6 patients identified as ‘positive’ by the AI model, all (100%) met HFpEF criteria. Among the 19 patients identified as ‘negative,’ 16 (84%) exceeded the PCWP/CO threshold. This yielded a sensitivity of 27%, specificity of 100%, PPV of 100%, NPV of 16%, positive likelihood ratio (LR+) of ∞, and negative likelihood ratio (LR−) of 0.73.

Among the 19 patients with resting haemodynamics only, 16 (84%) had elevated PCWP (≥ 15 mmHg). The AI model identified 11 patients as ‘positive,’ 10 of whom (91%) were true positives. Among the 8 ‘negative’ patients, 6 (75%) had elevated PCWP. This resulted in a sensitivity of 62%, specificity of 67%, PPV of 91%, and an NPV of 25%.

### Haemodynamic differences

**Figure 2:**
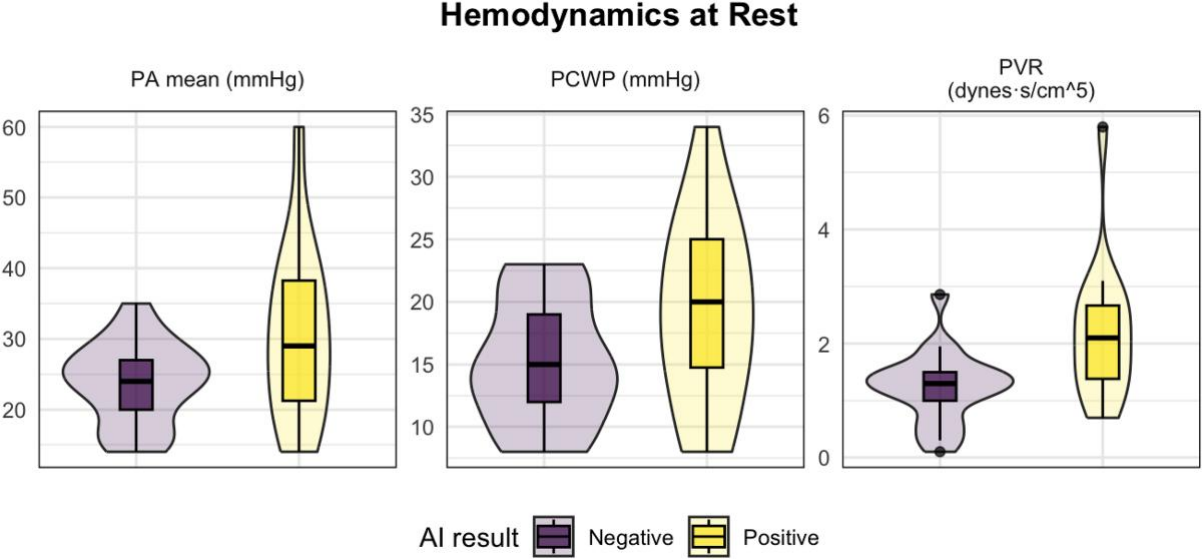
Box plots comparing haemodynamic data at rest between AI ‘positive’ and AI ‘negative’ cohorts. The box plots show the distribution of haemodynamic markers at rest that exhibit significant differences between the AI ‘Positive’ and AI ‘Negative’ cohorts, including PA mean (mmHg), PCWP (mmHg), and PVR (WU)

**Figure 3:**
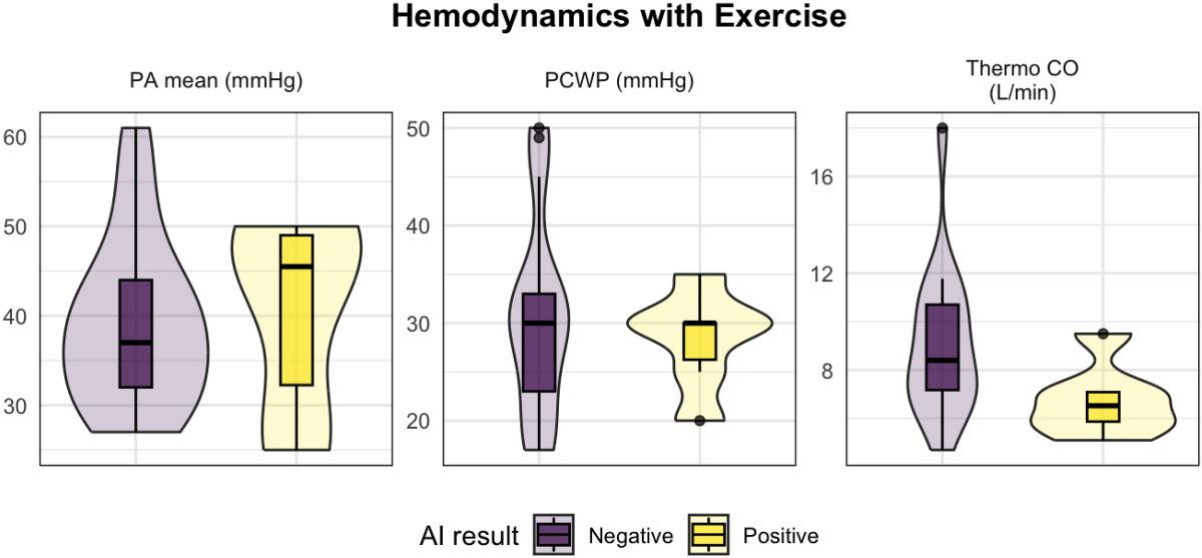
Box plots comparing haemodynamic data with exercise between AI ‘positive’ and AI ‘negative’ cohorts. The box plots display the distribution of haemodynamic markers during exercise for the AI ‘Positive’ and AI ‘Negative’ cohorts. It includes measurements of PA mean (mmHg), PCWP (mmHg), and cardiac output by thermodilution (L/min)

At rest, patients who screened positive by the AI tool had significantly higher pulmonary capillary wedge pressure (PCWP; median 20 vs 15 mmHg, *P* = .029), mean pulmonary artery (PA) pressure (29 vs 24 mmHg, *P* = .02), and pulmonary vascular resistance (PVR; 2.1 vs 1.3 Wood units, *P* = .002) compared to those who screened negative (*Figure 2*). Following exercise, there was no difference in PCWP (30 vs 30 mmHg, *P* = .9) or mean PA pressure (46 vs 37 mmHg, *P* = .4), but AI-positive patients demonstrated significantly lower thermodilution-derived cardiac output (CO; 7.1 vs 9.2 L/min, *P* = .036) and a numerically higher, but not statistically significant, PA systolic pressure (60 vs 50 mmHg, *P* = .06) (*Figure 3*). Full rest and exercise haemodynamic data for both groups are presented in *Table 2*.

**Table 2:** Haemodynamic data at rest and with exercise of patients in the full cohort and AI ‘positive’ and ‘negative’ cohorts

Baseline haemodynamics	Full cohort (*n* = 47)	AI positive cohort (*n* = 18)	AI negative cohort (*n* = 29)	*P*-value
**RA (mmHg)**	9.5	10	9	.40
**RV systolic (mmHg)**	39.5	47	35	.**02**
**RV diastolic (mmHg)**	10	10	10	.50
**PA systolic (mmHg)**	38	47.5	35	**<**.**01**
**PA diastolic (mmHg)**	20	23.5	16	**<**.**01**
**PA mean (mmHg)**	25	29	23.5	.**02**
**PCWP (mmHg)**	15	20	15	.**03**
**Fick CO (L/min)**	7.0	6.9	7.1	.50
**Fick CI (L/min/m^2^)**	3.4	3.6	3.3	.90
**Thermo CO (L/min)**	5.3	5.32	5.2	.50
**Thermo CI (L/min/m^2^)**	2.8	2.9	2.9	.85
**SVR (dyn·s/cm^5^)**	1010	1016	966	.60
**PVR (dyn·s/cm^5^)**	1.5	2.1	1.3	**<**.**01**
**SBP (mmHg)**	135	139	129	.20
**DBP (mmHg)**	74	72	74.5	.60
**MAP (mmHg)**	100	102	98	.30
**SVR (dyn·s·cm^−5^)**	1270	1328	1251	.50

Exercise haemodynamics	Full cohort (*n* = 28)	AI positive cohort (*n* = 7)	AI negative cohort (*n* = 21)	*P*-value
**RA (mmHg)**	16	17	16	.70
**PA systolic (mmHg)**	53	60	50	.06
**PA diastolic (mmHg)**	28	30	25	.90
**PA mean (mmHg)**	37	45.5	37	.40
**PCWP (mmHg)**	30	30	30	.90
**Thermo CO (L/min)**	7.5 (*n* = 27)	7.1	9.2 (*n* = 20)	.**03**
**Thermo CI (L/min/m^2^)**	4.2 (*n* = 27)	3.9	4.5 (*n* = 20)	.20
**SBP (mmHg)**	155	155	159	.50
**DBP (mmHg)**	81	68	85.5	.40
**PCWP:CO ratio**	4.47 (*n* = 27)	4.88	4.41 (*n* = 19)	.21
**SVR (dyn·s·cm^−5^)**	945 (*n* = 11)	1226	707 (n = 3)	.11

Values are presented as median or *n* (%). The *P*-values reported are based on a Wilcoxon test for non-normally distributed continuous variables. Bolded values indicate statistical significance at P < 0.05.

CO, cardiac output; CI, cardiac index; DBP, diastolic blood pressure; MAP, mean arterial pressure; PA, pulmonary artery; PCWP, pulmonary capillary wedge pressure; PVR, pulmonary vascular resistance; RA, right atrium; RV, right ventricular; SBP, systolic blood pressure; SVR, systemic vascular resistance; Thermo, thermodilution.

### Indeterminate HFpEF risk scores

**Figure 4:**
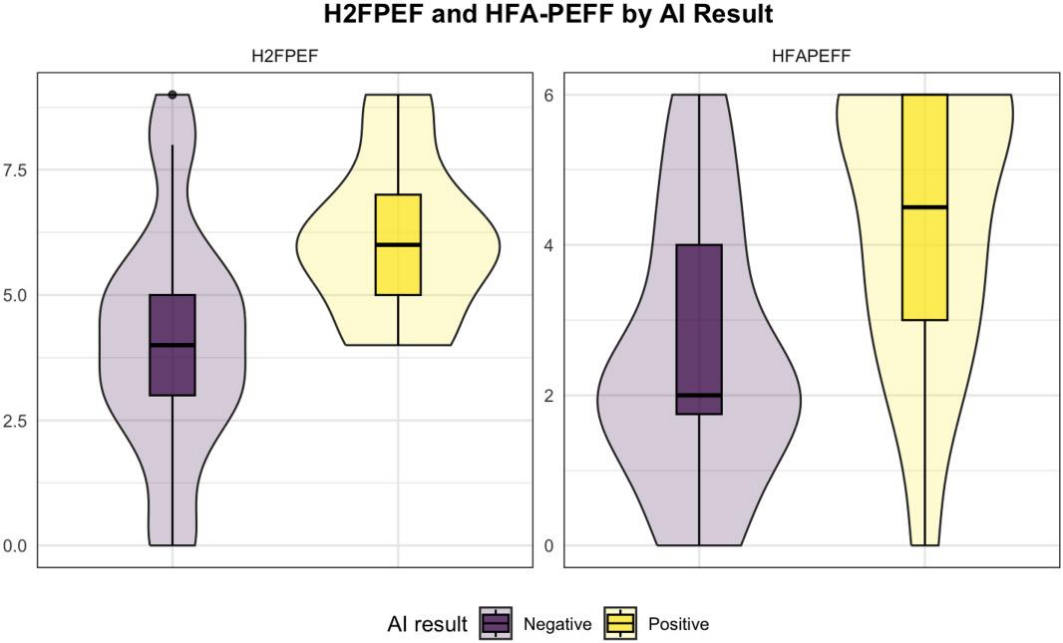
Box plots comparing the H2FPEF score and HFA-PEFF score. The box plots show the distribution of H_2_FPEF and HFA-PEFF scores for the AI ‘Positive’ and AI ‘Negative’ cohorts

Among the 47 patients in our cohort, 25 (53%) had H_2_FPEF scores in the indeterminate range (*Figure 4*).^2–5^ Of these 25 patients, 18 (72%) were ultimately diagnosed with HFpEF based on invasive haemodynamics. The AI model identified 5 patients as positive and 20 as negative. Among those who screened positive, 4 (80%) met invasive criteria for HFpEF. In contrast, 14 of the 20 patients who screened negative (70%) were also found to have HFpEF on haemodynamic assessment.

### TTE-RHC interval analysis

To assess the impact of time between TTE and RHC, we conducted a sensitivity analysis limited to patients with TTE-RHC intervals of <365 days (*n* = 33) and <90 days (*n* = 22). Across both timeframes, key resting haemodynamic differences between AI-positive and AI-negative patients were preserved. Resting PVR remained significantly higher in AI-positive patients in the full cohort and the <365-day group (2.2 vs 1.35 WU, *P* = .005), with a similar trend in the <90-day group (2.1 vs 1.44 WU, *P* = .069). Resting mean PA pressure was also significantly higher in AI-positive patients in both the <365-day (31 vs 22.5 mmHg, *P* = .011) and <90-day (30 vs 22.5 mmHg, *P* = .028) cohorts. Resting PCWP consistently differed between groups as well: <365 days (20 vs 14 mmHg, *P* = .023) and <90 days (20 vs 14 mmHg, *P* = .098). Differences in exercise haemodynamics trended in the expected direction but did not reach statistical significance, likely due to smaller sample sizes (see Supplementary Table S1).

## Discussion

This study externally validates a novel FDA-approved echocardiographic AI model for HFpEF using invasive haemodynamic testing, the gold standard for HFpEF diagnosis. Our findings reinforce the model’s high specificity and PPV when evaluated against invasive exercise haemodynamic criteria. To the best of our knowledge, this is the first study to assess the diagnostic performance of this AI tool using RHC data, the standard for HFpEF diagnosis in a small, high-prevalence referral cohort. While specificity was high, sensitivity was more modest, meaning that a positive result can add confidence, but a negative result should not be used to exclude HFpEF. Examining performance in this enriched referral population is important, as these are the contexts where diagnostic uncertainty is common and where AI tools may ultimately need to be refined and adapted.

Among patients who underwent invasive testing, the AI screen demonstrated excellent rule-in characteristics, with specificity ranging from 88% using standard exercise PCWP thresholds to 100% using the PCWP/CO slope criterion. Although subgroup sizes were modest (*n* = 28 for exercise RHC; *n* = 25 for indeterminate H_2_FPEF scores), its performance was preserved even among patients with indeterminate H_2_FPEF scores, a subgroup that comprised over half the cohort. In this grey zone, where clinical scores often fail to guide decision-making, the AI tool correctly identified 80% of those ultimately diagnosed with HFpEF, but it also missed a substantial proportion of true cases. Given the modest subgroup sizes, these results should be interpreted with caution. They suggest that the tool may provide rule-in value in specific contexts but may not be reliable as a stand-alone test.

At the cohort level, this pattern was even more apparent: high specificity but modest sensitivity and NPV. In this high-prevalence referral cohort, sensitivity ranged from 27% to –62% and NPV from 16% to 33%, underscoring the inability of non-invasive tools to reliably exclude HFpEF, particularly in early disease or borderline presentations. These findings reinforce the continued need for consideration of invasive testing in patients with unexplained dyspnoea and persistent clinical suspicion, regardless of negative non-invasive screen results.

Importantly, the model’s performance remained stable across a wide range of TTE-to-RHC intervals. Haemodynamic differences between AI-positive and AI-negative patients, including PCWP, mean PA pressure, and PVR, were consistent in both <365-day and <90-day sensitivity analyses. This suggests the model may retain relevance even when imaging and invasive testing are not performed concurrently, supporting its potential use in routine outpatient workflows.

Interestingly, the AI tool also appeared to identify patients with more advanced haemodynamic derangements. AI-positive individuals had significantly higher resting PCWP and PVR, as well as lower CO with exercise, findings consistent with more advanced disease. Consistent with the pulmonary vascular abnormalities seen with more advanced HFpEF, AI-positive individuals had a numerically, but not statistically significant, higher PA systolic pressure (60 vs 50 mmHg, *P* = .06). Intuitively, a model that detects abnormalities from resting echocardiography alone may be capturing patients with elevated filling pressures and worse physiology. Identifying these high-risk individuals is clinically meaningful, as they are more likely to experience adverse outcomes and may benefit from earlier intervention or closer follow-up.^20,21^ In this context, the tool’s strong PPV and specificity suggest utility not only for diagnosis, but for identifying patients with more severe HFpEF.

While both the AI tool and the H_2_FPEF score aim to improve non-invasive diagnosis, their combined use adds little in indeterminate cases, where each still misses subtle physiologic abnormalities. The AI model may offer practical advantages by relying solely on echocardiographic video data, eliminating the need for subjective or inconsistently available clinical inputs. Stacking imperfect tools may falsely suggest diagnostic clarity without improving accuracy. In such cases, clinicians may be better served proceeding directly to invasive testing, which remains the diagnostic gold standard. Ultimately, this model may be best suited as a screening tool in broader, low prevalence, populations, finding individuals who warrant cardiology referral and further evaluation, rather than resolving ambiguity in already complex clinical scenarios.

### Limitations

This study has several limitations. First, it was conducted at a single academic centre, which may limit generalizability to broader clinical settings. The overall sample size was modest, particularly in subgroup analyses, which may reduce statistical power to detect certain differences. In addition, the patient population was predominantly Black (79%) and women (82%), which decreases generalizability. However, these groups are historically underrepresented in cardiology trials despite being disproportionately affected by HFpEF. The inclusion of this population provides important external validation in a high-prevalence, underrepresented group, and underscores that newly developed AI diagnostic tools require careful validation across diverse cohorts rather than assuming universal performance.^22–24^ While our cohort was enriched with patients warranting invasive exercise haemodynamics, a key strength, it may also reflect a higher pre-test probability of HFpEF than seen in community practice, potentially inflating positive predictive values. Five percent of patients had indeterminate AI results, and while this rate was low, especially compared to the rate of indeterminate H_2_FPEF scores, it underscores the need for standardized TTE protocols when utilizing this tool. Finally, although we used temporally closest TTEs for analysis, TTE-RHC intervals were variable, and disease progression between studies could influence concordance.

## Conclusion

In a real-world cohort referred for suspected HFpEF, an AI-based TTE tool demonstrated high specificity and PPV compared against invasive haemodynamic criteria. The algorithm correctly reclassified nearly half of patients with indeterminate H_2_FPEF scores and maintained consistent performance regardless of the interval between TTE and RHC. However, sensitivity was modest, reinforcing that the tool may not be relied upon as a stand-alone rule-out test. Instead, it may provide added diagnostic value for identifying high-risk patients and guiding further evaluation. Given the modest sample size and the specific demographic characteristics of our cohort, these results should be interpreted cautiously and validated in larger, more diverse populations. More prospective validation is warranted to refine its clinical role in HFpEF diagnosis.

## Declarations

### Data Availability

The data underlying this study contain protected patient information and are not publicly available. De-identified data may be made available from the corresponding author upon reasonable request and with appropriate institutional approvals.
